# Solvent-Driven Chirality Switching of a Pillar[4]arene[1]quinone Having a Chiral Amine-Substituted Quinone Subunit

**DOI:** 10.3389/fchem.2021.713305

**Published:** 2021-07-07

**Authors:** Chunhong Liu, Zhipeng Yu, Jiabin Yao, Jiecheng Ji, Ting Zhao, Wanhua Wu, Cheng Yang

**Affiliations:** Key Laboratory of Green Chemistry and Technology of Ministry of Education, College of Chemistry, and Healthy Food Evaluation Research Center, Sichuan University, Chengdu, China

**Keywords:** pillar[4]arene[1]quinone, charge-transfer interaction, circular dichroism, anisotropy factors, chirality switching, solvent effects

## Abstract

Several new chiral pillar[4]arene[1]quinone derivatives were synthesized by reacting pillar[4]arene[1]quinone (**EtP4Q1**), containing four 1,4-diethoxybenzene units and one benzoquinone unit, with various chiral amines *via* Michael addition. Due to the direct introduction of chiral substituents on the rim of pillar[n]arene and the close location of the chiral center to the rim of **EtP4Q1**, the newly prepared compounds showed unique chiroptical properties without complicated chiral resolution processes, and unprecedented high anisotropy factor of up to −0.018 at the charge transfer absorption band was observed. Intriguingly, the benzene sidearm attached pillar[4]arene[1]quinone derivative **1a** showed solvent- and complexation-driven chirality inversion. This work provides a promising potential for absolute asymmetric synthesis of pillararene-based derivatives.

## Introduction

Manipulating molecular chirality, being in the core position of contemporary chemical science (Zhang et al., 2014), has been attracting significant attention not only from the point of view of the fundamental science but also the potential applications such as chiral recognition, asymmetry catalysis, and chiral switches (Zhou and Tang, 2005; Goldup, 2016; Gao et al., 2017; Xing and Zhao, 2018; Corra et al., 2019; Yao et al., 2021b). On the other hand, supramolecular chiral photochemistry, which arises from the chiral spatial arrangement of noncovalently involved components in assemblies (Crassous, 2009), has received booming development in recent years due to their close correlation with many natural and artificial systems and a wide range of potential applications (Jung et al., 2001; Nakashima et al., 2001; Borovkov et al., 2003a; Hembury et al., 2008; Yang and Inoue, 2014; Chen et al., 2015; Liu et al., 2015; Wang X. et al., 2020). Compared with molecular chirality, the supramolecular chirality is more attractive in terms of their regulatability by the external conditions such as temperature (Yao et al., 2017; Fan et al., 2019), pH (Kanagaraj et al., 2020; Liang et al., 2020; Hao et al., 2021), redox (Xiao et al., 2020), light (De Poli et al., 2016), chemical additives (Lee et al., 2018), pressure (Yao et al., 2021a), and solvents (Borovkov et al., 2003b; Fan et al., 2019). Pillar[n]arenes (Ogoshi et al., 2008; Xue et al., 2012; Pan et al., 2015; Fan et al., 2016; Jie et al., 2018; Lv et al., 2018; Li G. et al., 2019; Xiao et al., 2019; Ji et al., 2020a; Lou and Yang, 2020; Mi et al., 2020; Liu et al., 2021; Peng et al., 2021), as a relatively new class of synthetic macrocyclic hosts with some unique properties (Guo et al., (2018); Lai et al., (2019); Liu et al., (2019); Wang et al., (2021), have proved to be an ideal platform to construct unimolecular chirality based on different external stimuli-driven. We have demonstrated that the chirality of pillar[n]arene derivatives could be manipulated by external stimuli, including temperature, redox, light, and pressure (Yao et al., 2017; Xiao et al., 2020; Yao et al., 2021a; Yao et al., 2021b). The synthetic approaches for obtaining chiral pillar[5]arenes include introducing chiral or bulky groups on the openings, fusing a side ring onto one subunit, or threading with an axle to block the interconversion between *S*_*p*_ and *R*_*p*_ conformers (Ogoshi et al., 2011; Chen et al., 2013; Strutt et al., 2014b; Shurpik et al., 2016; Li Q. et al., 2019; Ma et al., 2019; Zhang et al., 2019). However, pillar[5]arenes’ planar-chiral *S*_*p*_ and *R*_*p*_ enantiomers need to be separated by HPLC enantio-resolution of the racemic mixture to study their chiroptical properties. Synthesis of chiral pillar[5]arenes without the complicated chiral resolution processes should be more convenient and valuable for studying supramolecular chirality switching. It has been reported that 1,4-benzoquinone undergoes the Michael addition reaction with aliphatic or aromatic amines to selectively afford 2,5-bis(alkyl/arylamino)-1,4-benzoquinones (Almeida Barbosa et al., 2010; Strutt et al., 2014a; Li et al., 2018; Kiruthika et al., 2020; Li et al., 2020). In this work, we report the synthesis of several new chiral pillar[4]arene[1]quinone derivatives and their unique chiroptical properties. We report the synthesis of several new pillar[4]arene[1]quinone derivatives by attaching chiral amines onto the quinone ring of **EtP4Q1**. Homochiral compounds were obtained in moderate to good separate yield without complicated HPLC chiral resolution. These compounds showed unique chiroptical properties with unprecedented high anisotropy *g* factor of up to −0.018 at the charge transfer absorption band; moreover, the benzene sidearm attached pillar[4]arene[1]quinone derivative **1a** showed solvent- and complexation-driven chirality inversion.

## Experiment

### Compounds

**SCHEME 1:**
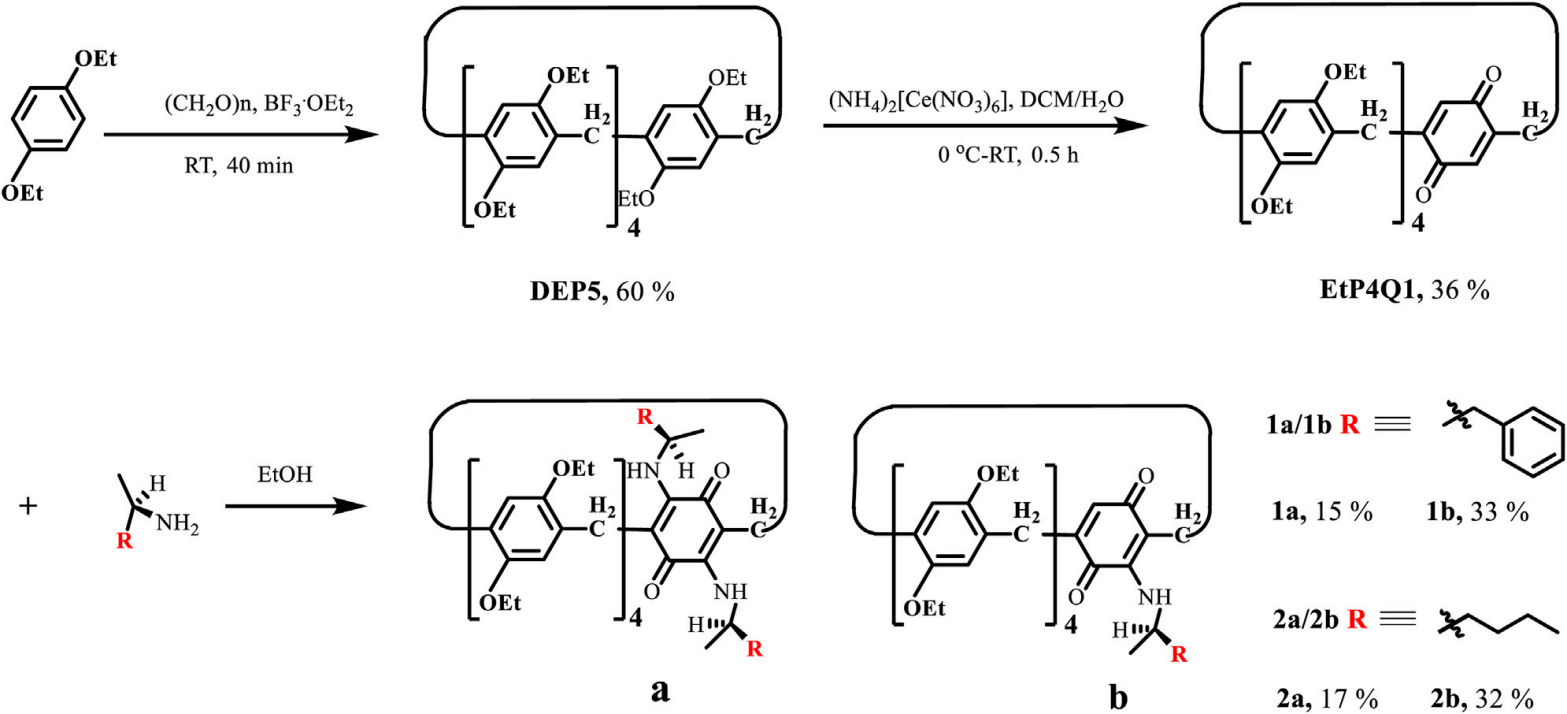
Synthetic routes of chiral amine-bearing pillar[4]arene[1]quinones.

A general reaction scheme for the synthesis of chiral pillar[4]arene[1]quinone derivatives is shown in Scheme 1. Diethyl hydroquinone ether–based pillar[5]arene (**DEP5**) was synthesized according to the literature procedure (Ogoshi et al., 2010). Pillar[4]arene[1]quinone (**EtP4Q1**), in which a benzoquinone unit replaces a diethoxybenzene unit in **DEP5**, was synthesized by partial oxidation with ammonium cerium nitrate, following a modified version of the literature procedure (Han et al., 2012). The reactions of the achiral **EtP4Q1** with chiral amines were carried out in ethanol at 75°C in an oil bath for 24 h (Almeida Barbosa et al., 2010; Li et al., 2018). After the solvent was removed under vacuum, the residue was purified by silica gel flash column chromatography using ethyl acetate/petroleum ether as the eluent to give the desired target product (see the supplementary file for detailed experimental procedures and characterizations).

### Materials and Instruments

Unless otherwise noted, all reagents and materials were commercially available and used without further purification. ^1^H NMR was recorded in a CDCl_3_ solution at room temperature on Bruker AMX-400 (operating at 400 MHz for ^1^HNMR), and all chemical shifts are reported in ppm with TMS as the internal standard. HRMS data were measured with a Waters Q-TOF Premier instrument. UV-vis spectra were obtained on a JASCO V650 spectrometer at room temperature. Circular dichroism spectra were recorded on a JASCO J-1500 spectrometer, and the obtained data were analyzed using ORIGIN 9.0 software.

## Results and Discussion

### Synthesis of 1a/1b and 2a/2b

1,4-Benzoquinones were known to undergo the Michael addition reaction with organic amines to give 2,5-bis(amino)-1,4-benzoquinones (Almeida Barbosa et al., 2010). Huang and coworkers demonstrated that pillar[4]arene[1]quinone could physically adsorb organic amines in the solid-state, which underwent *in situ* Michael addition by elevating the temperature to realize so-called solid–vapor post-synthetic modification (Li et al., 2018). In general, pillar[n]arene derivatives have a pair of planar chiral enantiomeric conformers, which could interconvert through the “oxygen-through-the-annulus” rotation. The attachment of bulky groups on the rims of pillar[n]arene could block the interconversion and lead to a pair of separable enantiomers. The same could be realized by introducing a side ring or threading an axle. Enantiopure pillar[n]arene derivatives showed extremely strong chiroptical properties at the absorption band of hydroquinone ethers due to the inter-ring unit exciton coupling effect. Direct introduction of chiral substituents on the rim of pillar[n]arene could also lead to chiral pillar[n]arene derivatives (Ogoshi et al., 2011; Strutt et al., 2012; Chen et al., 2013; Strutt et al., 2014b; Shurpik et al., 2016; Li Q. et al., 2019; Ma et al., 2019; Zhang et al., 2019). However, the chiral substituents are far away from the aromatic rings in distances and usually show weak chiroptical induction. **EtP4Q1** showed brown charge transfer absorption. The Michael addition reaction allows chiral amines to be introduced onto the quinone ring directly, and we envisioned that the chiral **EtP4Q1** should offer unique chiroptical properties differing from other chiral pillar[5]arene derivatives. **EtP4Q1** was reacted with chiral (R)-(+)-α-methylbenzylamine (Scheme 1) in ethanol, which led to two brown products in 15 and 33% yields, respectively, which were demonstrated to be the mono- (**1a**) and di-substituted (**1b**) products, respectively, based on the NMR and HRMS analyses. The same was true in the reaction of (R)-2-aminohexane, which gave the mono- and di-substituted products **2a** and **2b**, respectively, after the silica gel chromatography separation.

### UV-Vis Spectral Studies

**FIGURE 1:**
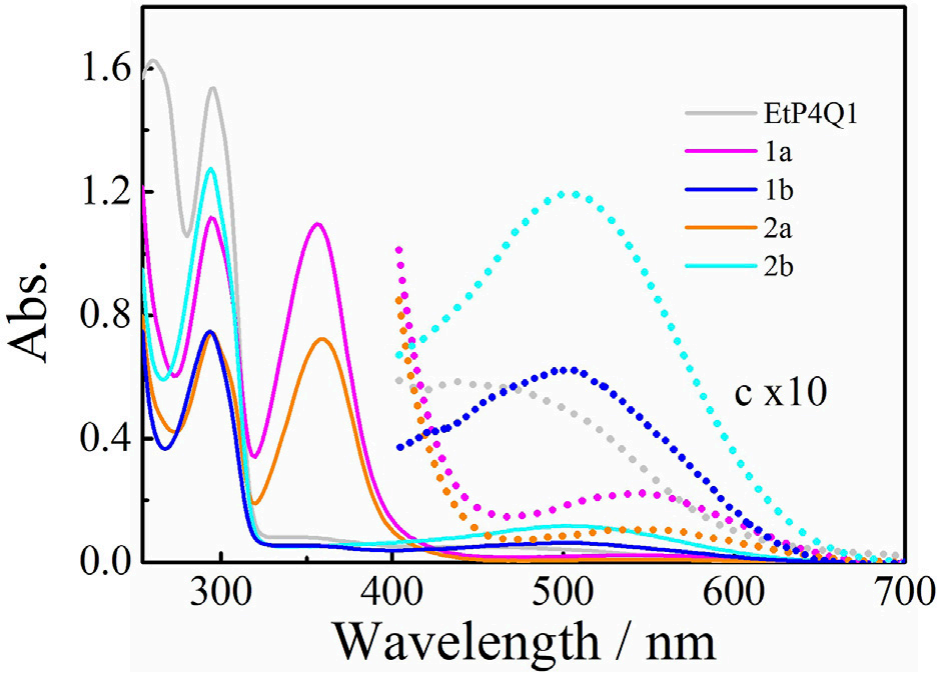
UV-vis absorption spectra of **EtP4Q1**-derivated compounds in chloroform solution (50 μM).

The UV-vis spectra of chiral amine-substituted pillar[4]arene[1]quinones were measured in chloroform at 25°C. **EtP4Q1** showed a sharp absorption peak at 294 nm and a broad absorption at the visible range (Figure 1), assignable to the transitions of hydroquinone ether units and the intramolecular charge transfer, respectively (Mi et al., 2020; Mi et al., 2021). The UV-vis spectra of the mono-substituted pillar[4]arene[1]quinone derivatives **1b** and **2b** exhibited two major transitions, showing a weak broad absorption that tailed to 400–700 nm, which is assignable to a CT transition. Similar to that of **EtP4Q1,** the strong absorption that peaked at ca. 300 nm could be ascribed to the absorption of the hydroquinone units. Interestingly, **1a** and **2a** showed two intensive peaks in the UV range and a broad absorption at 450–700 nm that is bathochromic shifted with a concomitant decrease in intensity compared to those of **1b** and **2b**. The attenuated CT interaction of **1a** and **2a** could be presumably ascribed to the reduced electron withdraw property of the benzoquinone ring when substituted with two amino substituents, which weakened the intramolecular CT interactions. An independent spectral titration for the intermolecular complexation between **EtP4Q1** and (R)-(+)-α-methylbenzylamine, by increasing the concentration of (R)-(+)-α-methylbenzylamine, was carried out in CHL at 25°C (ESI, Supplementary Figure S13). It turned out that the addition of (R)-(+)-α-methylbenzylamine to a solution of **EtP4Q1** did not lead to new absorption in the wavelengths range of 300–400 nm and visible region in the UV-vis spectroscopy, demonstrating that the new absorptions originated from the conjugation of the chiral amine-substituent in the quinone ring.

**FIGURE 2:**
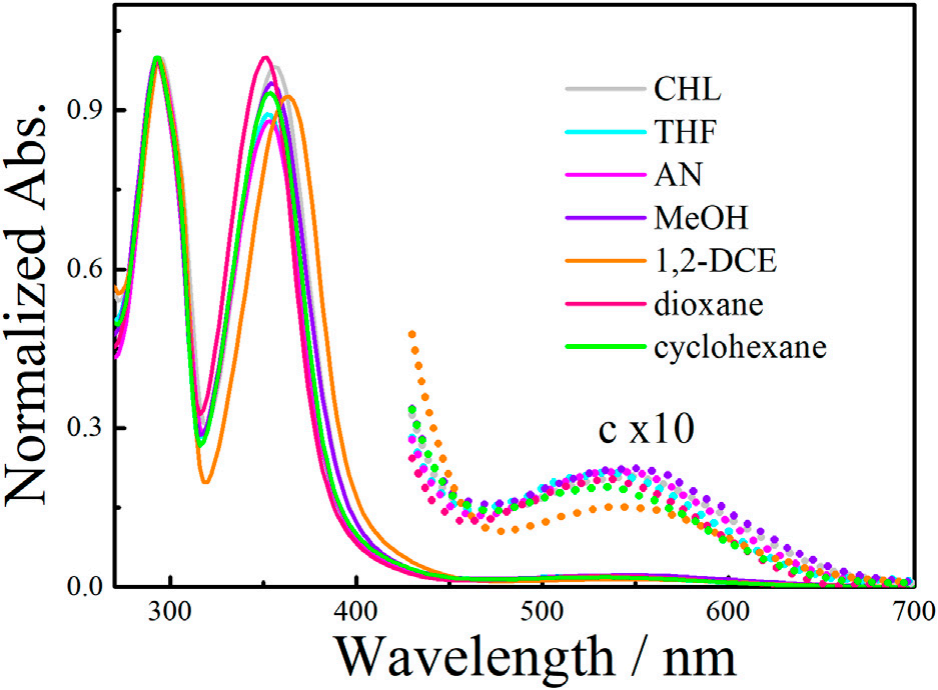
Normalized UV-vis absorption spectra of **1a** compound in various solvents (50 μM).

Weak ground-state intermolecular interactions, including CT, should be significantly affected by environmental factors such as solvent polarity, temperature, and so on (Saito et al., 2004). We have demonstrated that intermolecular supramolecular interactions can be effectively manipulated by adjusting the environmental effectors, including temperature or solvent (Ji et al., 2020b). As illustrated in Figure 2, the solvent-dependent UV-vis absorption spectra of **1a** revealed the CT transition was not restricted to chloroform solution but rather could be observed in various solvents. Moreover, the absorption spectra of the **1a** (50 μM), being measured in chloroform at various temperatures (Supplementary Figure S14), showed inconspicuous temperature-dependent behavior of the CT band, confirming that the intramolecular CT dominate in the macrocyclic structure.

### Chiroptical Properties of the Macrocyclic Compounds

**FIGURE 3:**
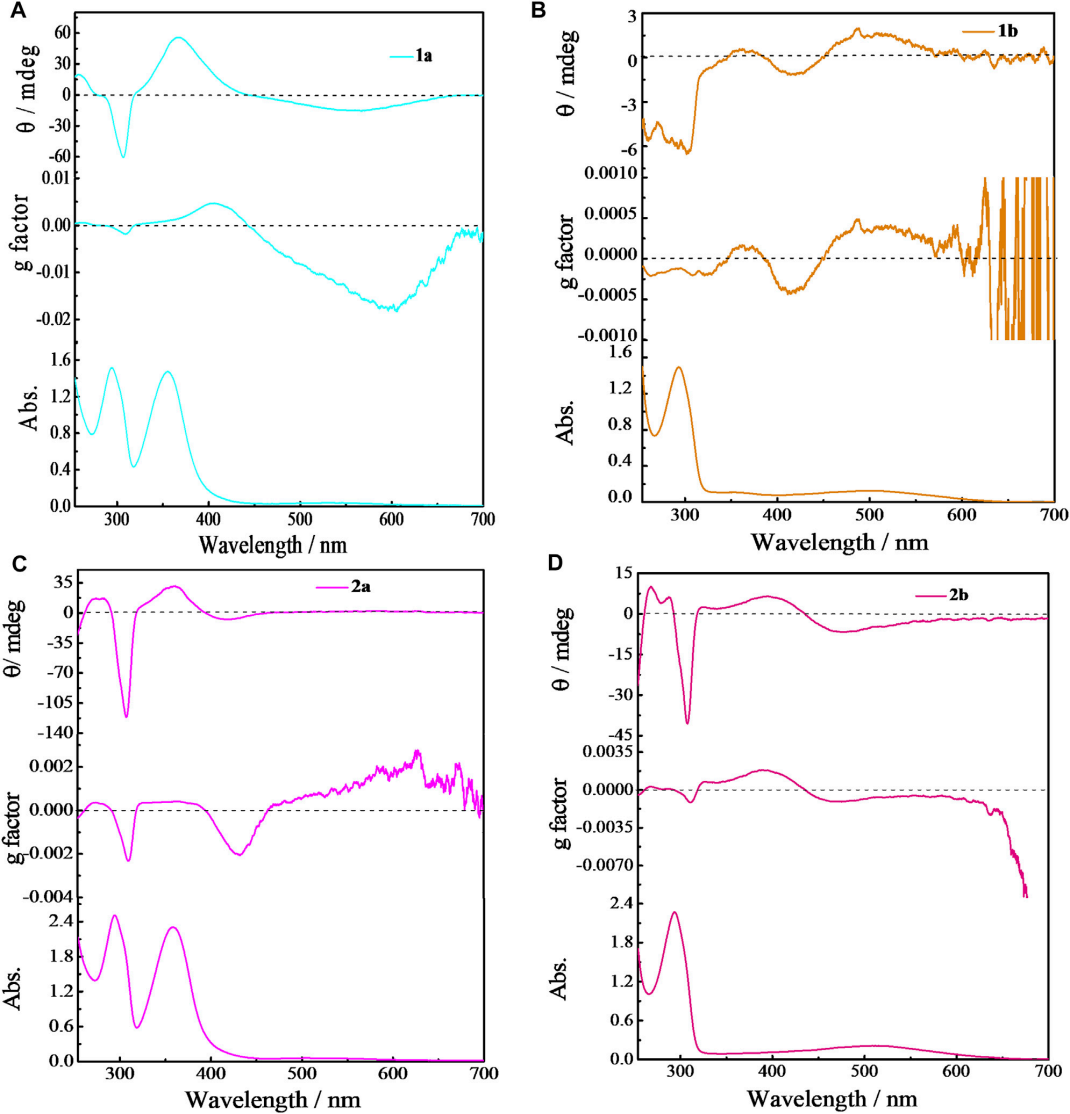
Circular dichroism and UV-vis absorption spectra of **EtP4Q1-**derivated compounds (100 μM) in CHL solution **(A) 1a**; **(B) 1b**; **(C) 2a**; **(D) 2b**.

As mentioned above, pillar[5]arene derivatives possess a pair of enantiomeric conformers, and in general, they adapt per-*R*_*p*_ (*R*_*p*_, *R*_*p*_, *R*_*p*_, *R*_*p*_, and *R*_*p*_) or per-*S*_*p*_ (*S*_*p*_, *S*_*p*_, *S*_*p*_, *S*_*p*_, and *S*_*p*_) configurations to avoid inter-subunit steric repulsion. We have demonstrated that *R*_*p*_ and *S*_*p*_ conformers gave intensive positive and negative circular dichromism (CD) signals, respectively, at the extrema around 310 nm. The *R*_*p*_ and *S*_*p*_ conformers usually have an equal population (Yao et al., 2017; Xiao et al., 2020). Such conformational equilibrium could be broken by the complexation of a chiral guest to induce CD response, and thus being applied to chiral sensing (Ji et al., 2020a; Chen et al., 2020). In the chiral amine-substituted **EtP4Q1** derivatives, the chiral aliphatic amine or aromatic amine is anchored on the quinone subunit, with the chiral center located close to the rim of **EtP4Q1**, which was expected to significantly influence the chiroptical properties. CD spectra of **1a**/**1b** and **2a**/**2b** were measured at 25°C in chloroform to study the chiroptical properties (Figure 3). Negative Cotton effects at around 300 nm were observed for **1a**, **1b**, **2a**, and **2b,** assignable to the π-π* transition of hydroquinone units, which indicated that the hydroquinone units arranged in *S*_*p*_ configurations in the presence of the chiral amine group.

Despite the CD signals at 310 nm, new Cotton effects appeared in the region of 300–400 nm of **1a** and **2a**, which could be ascribed to the transition of 2,5-bis(alkyl/arylamino)-1,4-benzoquinones (Martini and Nachod, 1951; Li et al., 2020). In addition, strong Cotton effects in CT transition in the wavelength region of 400–700 nm were observed (Wang H. J. et al., 2020). In particular, the *g* factor of up to −0.018 was observed with **1a**, which, to our knowledge, is the largest *g* factor ever reported for CT transition (Mori and Inoue, 2005; Mori et al., 2006).

**FIGURE 4:**
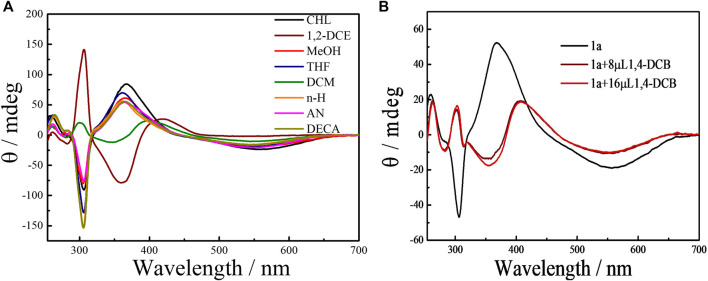
**(A)** CD spectra of **1a** (50 μM) in various solvents at 25°C. Key: n-H, *n*-hexane; DECA, decalin; DCM, dichloromethane; THF, tetrahydrofuran; 1,2-DCE, 1,2-dichloroethane; CHL, chloroform; AN, acetonitrile and MeOH, methanol. **(B)** CD spectra of **1a** (50 μM) in chloroform solvents by adding 1,4-dicyanobutane (1,4-DCB) at 25°C.

The effect of solvents on the planar chirality of **1a** was investigated. We have demonstrated that negative CD extrema at ca. 310 nm corresponds to *S*_*p*_ configuration of pillar[n]arenes, and vice versa for the *R*_*p*_ configuration(Yao et al., 2017; Xiao et al., 2020). The strong CD spectra observed with these chiral **EtP4Q1** derivatives suggested an unequal population of chiral conformers. We expected that variation of environmental conditions might switch the equilibrium of conformers and thus cause chiroptical change. Indeed, **1a** exhibits negative CD_ex_ in most of the solvents examined, including hexane, acetonitrile, decahydronaphthalene, chloroform, methanol, and THF (Figure 4A), suggesting the *S*_*p*_ configuration dominate in these solvents. However, the CD_ex_ at ca. 300 nm was inverted in sign accompanying by a hypochromic shift to give positive CD_ex_ in 1,2-dichloroethane and dichloromethane, indicating inversion of planar chirality to *R*_*p*_. This result revealed that the relative stability between diastereomeric conformers could be significantly changed by the solvent. The following two aspects were responsible for the chiroptical switching process. The solvation of the chiral amine will cause significant steric interaction between solvent molecules surrounding the chiral amine substituents and hydroquinone ether units to thus critically affect the chiral arrangement of hydroquinone subunits. Also, DCM and 1,2-DCE were known to complex with pillar[5]arene derivatives, which will push the sidearms of the chiral amine towards the outside of the cavity. Indeed, NMR titration experiments of compound **1a** in CDCl_3_ upon adding different potions of 1,2-DCE showed that the proton signals of chiral amine significantly shifted downfield and the aromatic protons in pillar[5]arene become broad first and then separated into multiple peaks, when added more than 8% 1,2-DCE (Supplementary Figure S26). The chiral center that is closely located at the opening of the macrocyclic ring played an important role in the chiral inversion behavior. Solvent-dependent chiroptical changes were also observed with **1b** (Supplementary Figure S20).

The complexation-driven chiral optical switching has also been observed with bicyclic pillar[5]arene derivatives due to the exclusion of the side ring by the complexation of a guest molecule (Yao et al., 2017; Fan et al., 2019; Xiao et al., 2020). We found that stereoinversion with a sign-switching of CD_ex_ from negative to positive was induced by the addition of 1,4-dicyanobutane (1,4-DCB), a strong P[5] cavity binder (Shu et al., 2012), to **1a** in chloroform (Figure 4B; Supplementary Figures S24, S25).

This is consistent with the chiral inversion phenomenon observed in the solvents of DCM and 1,2-DCE, further suggesting that the benzene ring of the chiral amine is located toward the inside of the cavity. The bulky and rigid benzene ring should cause significant steric repulsion with the complexed 1,4-DCB, when directing inside the cavity to lead to conformational inversion. This conclusion could be supported by the fact that the originally negative CD_ex_ intensity in dichloromethane was further enhanced rather than inverted upon the gradual addition of 1,4-DCB to a solution of **1a** (Supplementary Figure S23). However, for **2a** and **2b**, which possess aliphatic sidearms, no solvent-/complexation-driven *S*_*p*_ to *R*_*p*_ chirality switching could be observed (ESI, Supplementary Figures S21, S22). We ascribe this to the flexible aliphatic sidearm in **2a**/**2b**, which will not bring significant steric interaction with the complexed guest/solvent molecules.

We have demonstrated that temperature variation could also cause chiroptical switching of bicyclic pillar[n]arenes due to the relatively large entropy changes between the self-included and self-excluded conformations. Variation temperature CD of **1a** was measured in different solvents, which, however, showed only the intensity’s variation to a certain extent (ESI, Supplementary Figures S16–S19) while the CD sign was never inverted. Similar was true with other chiral **EtP4Q1** derivatives, suggesting a small entropy difference between diastereomeric conformers.

## Conclusion

In summary, we synthesized a series of new chiral amines functionalized pillar[4]arene[1]quinones, which showed unique chiroptical properties. In particular, **1a** showed strong CD signals at the CT absorption band with an unprecedented high anisotropy *g* factor of up to −0.018. Interestingly, we found that the pillar[4]arene[1]quinone having a benzene sidearm showed solvent- and complexation-driven chirality inversion, while no chirality inversion could be observed with the analogs having aliphatic sidearm. The present results opened a new window for synthesizing pillar[n]arene-based stimuli-responsive chiral molecular devices and provide a promising potential for absolute asymmetric synthesis of pillararene-based derivatives.

## Data Availability

The original contributions presented in the study are included in the article/Supplementary Material; further inquiries can be directed to the corresponding authors.
