# Supplementary material for: Solvent-Driven Chirality Switching of a Pillar[4]arene[1]quinone Having a Chiral Amine-Substituted Quinone Subunit
Source: Front Chem. 2021 Jul 7;9:713305. doi: 10.3389/fchem.2021.713305 (PMC8293272; doi:10.3389/fchem.2021.713305)
Supplement: Supplementary file 1 [file DataSheet1.pdf]

Supplementary Material

Solvent-Driven Chirality Switching of a Pillar[4]arene[1]quinone having a Chiral Amine-substituted Quinone Subunit

Chunhong Liu [1], Zhipeng Yu[1], Jiabin Yao[1], Jiecheng Ji[1], Ting Zhao[1], Wanhua Wu[1*], Cheng Yang [1*]

[1]Key Laboratory of Green Chemistry & Technology of Ministry of Education, College of Chemistry, State Key Laboratory of Biotherapy, and Healthy Food Evaluation Research Center, Sichuan University, Chengdu 610064, China.

Table of contents

**1.** Synthesis and Characterization	**P3**
**2.** UV-vis spectra of **1a**	**P11**
**3.** Variable Temperature CD Spectra of **1a**	**P12**
**4.** Solvent-dependent CD Spectra of **1b, 2a** and **2b**	**P15**
**5.** Spectroscopic studies of host and guest binding complexes	**P16**
**6.** Reference	**P18**

Synthesis and Characterization

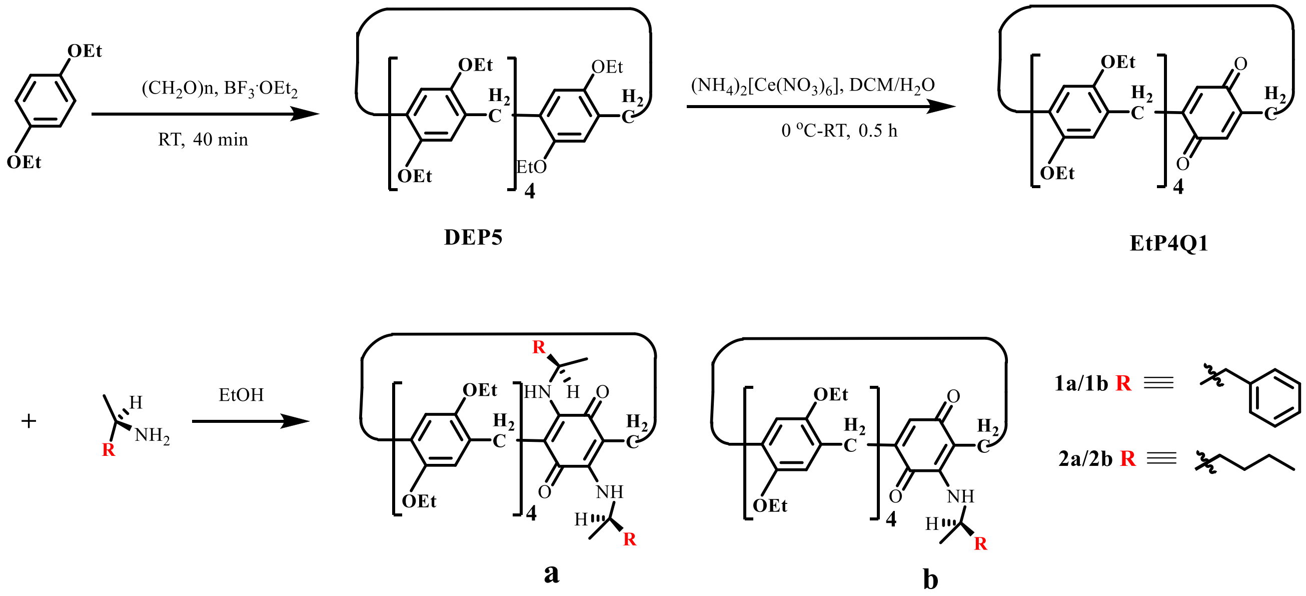

Scheme S1. Synthetic routes of chiral amine-substituted pillar[4]arene[1]quinones.

Compound **DEP5** and **EtP4Q1** were prepared according to the previous reports(Ogoshi et al., 2010; Han et al., 2012)

The synthesis of compound 1a/1b: **EtP4Q1** (150 mg, 0.18 mmol) and EtOH 98% (v/v) (1 mL) were placed in a Schlenk tube, then (R)-(+)-α-methylbenzylamine (138.0 μL, 1.08 mmol) was added slowly to the solution. The mixture was stirred at 75 °C in an oil bath for 24 h until the complete consumption of the starting material (TLC analysis). The solvent was removed under reduced pressure, and the residue was purified by silica gel column chromatography eluting with 10:1 (v/v) petroleum ether /EA to give the desired compounds as red solid.

1a: 1a was obtained in 15 % separation yield. ^1H NMR spectra for **1a** was shown in Figure S1: ^1H NMR (400 MHz, $CDCl_3$) δ 7.28-7.00 (m, 10H), 6.88 (s, 2H), 6.68-6.52 (m, 6H), 5.37 (dd, J = 12.4, 5.9 Hz, 2H), 3.92-3.38 (m, 26H), 1.42 (s, 4H), 1.33 (t, J = 6.9 Hz, 6H), 1.29 -1.22 (m, 8H), 1.16 -1.05 (m, 12H). ^{13}C NMR (100 MHz, $CDCl_3$) δ 181.3, 150.4, 150.3-150.2, 149.8, 144.5, 130.1-128.6, 128.3, 127.6-127.5, 127.6-124.6, 115.3, 64.3-63.7, 53.2, 30.6, 29.8, 15.4, 15.2-14.7. ESI-MS of **1a** is shown in Figure S3: HRMS (ESI) *m/z* : $[M+H]^+$, Calcd for $C_{67}H_{79}N_2O_{10}$, 1071.5729; Found 1071.5736;

1b: **1b** was obtained in 33% separation yield. ^{1}H NMR spectra for **1b** was shown in Figure S4: 1H NMR (400 MHz, $CDCl_3$) δ 7.05-6.85 (m, 6H), 6.85-6.80 (m, 3H), 6.73 (d, J = 6.8 Hz, 2H), 6.59 (s, 1H), 6.48 (d, J = 4.9 Hz, 2H), 5.25 (p, J = 6.7 Hz, 1H), 3.98-3.70 (m, 24H), 3.65 (d, J = 1.3 Hz, 1H), 3.47 (s, 1H), 1.41 (d, J = 3.2 Hz, 2H), 1.39 (d, J = 3.3 Hz, 4H), 1.37 (s, 3H), 1.35 (s, 2H), 1.33 (d, J = 3.6 Hz, 1H), 1.24 (dt, J = 10.7, 3.5 Hz, 12H), 1.18 (t, J = 7.0 Hz, 3H). ^{13}C NMR (100 MHz, $CDCl_3$) δ 186.1, 185.8, 151.6, 150.4, 149.9, 149.7, 149.5, 148.5, 144.9, 142.6, 134.1, 129.5, 128.9, 128.2, 127.1, 126.6, 126.3, 125.2, 123.1, 117.6, 115.9, 115.5, 115.2-114.8, 114.6, 114.2, 65.9, 63.9, 63.2, 53.7, 31.6, 29.2, 24.2, 21.7, 19.1, 15.4-14.6, 13.7 ppm. ESI-MS of **1b** is shown in Figure S6: HRMS (ESI) *m/*z : $[M+K]^+$, Calcd for $C_{59}H_{69}NO_{10}K$, 990.4553; Found 990.4529;

The synthesis of compound 2a/2b: In a Schlenk tube, **EtP4Q1** (100 mg, 0.12 mmol) was dissolved in 1 mL EtOH 98% (v/v), then (R)-2-aminohexane (131.00 μL, 1.08 mmol) was added slowly. The mixture was stirred at 75 °C in an oil bath for 24 h until complete consumption of the starting material (TLC analysis). The solvent was removed under reduced pressure, and the residue was purified by silica gel column chromatography eluting with 20:1 (v/v) petroleum ether/EA to give the desired compounds as red solid.

2a: 2a was obtained in 17 % separation yield. ^{1}H NMR spectra for **2a** was shown in Figure S7: ^{1}H NMR (400 MHz, $CDCl_3$) δ 6.87 (s, 2H), 6.72 (d, *J* = 27.8 Hz, 4H), 6.60 (s, 2H), 4.18 (s, 2H), 3.95-3.59 (m, 26H), 1.60-0.76 (m, 48H).^{13}C NMR (100 MHz, $CDCl_3$) δ 180.9, 150.8, 150.0, 149.8, 149.1, 128.5, 127.8, 127.3, 116.1, 114.7 , 114.5 , 80.6-65.4 , 64.9 , 64.0 , 37.5 , 29.8-29.5 , 22.7 , 21.6 , 15.5-14.7 , 13.7 ppm. ESI-MS of **2a** is shown in Figure S9: HRMS (ESI) *m/*z : $[M+H]^+$, Calcd for $C_{63}H_{87}N_2O_{10}$, 1031.6355; Found 1031.6343;

2b: 2b was obtained in 32 % separation yield. ^{1}H NMR spectra for **2b** was shown in Figure S10: ^{1}H NMR (400 MHz, $CDCl_3$) δ 6.97 (d, *J* = 6.3 Hz, 2H), 6.86 (s, 1H), 6.81 (s, 1H), 6.77 (s, 1H), 6.74 (s, 1H), 6.70 (s, 1H), 6.45 (s, 1H), 6.39 (s, 1H), 4.09-3.86 (m, 12H), 3.83-3.70 (m, 12H), 3.68-3.51 (m, 3H), 1.54-1.37 (m, 20H), 1.34-1.10 (m, 10H), 0.98 (d, *J* = 3.9 Hz, 3H), 0.75 (dd, *J* = 101.4, 34.9 Hz, 3H).^{13}C NMR (100 MHz, $CDCl_3$) δ 187.2, 185.3, 152.2, 150.7, 150.3, 149.9, 149.6-149.3, 148.2, 143.6, 133.1, 130.1, 128.5-128.1, 126.3, 122.7, 117.9, 115.6-114.9, 114.4, 113.4, 66.1, 64.0, 63.7, 63.5-63.1, 62.9, 50.3, 38.7, 31.6, 29.7, 28.2, 26.8, 22.0, 20.7, 20.3, 15.2, 12.9 ppm. ESI-MS of **2b** is shown in Figure S12: HRMS (ESI) *m/*z : $[M+H]^+$, Calcd for $C_{57}H_{74}NO_{10}$, 932.5307; Found 932.5285;

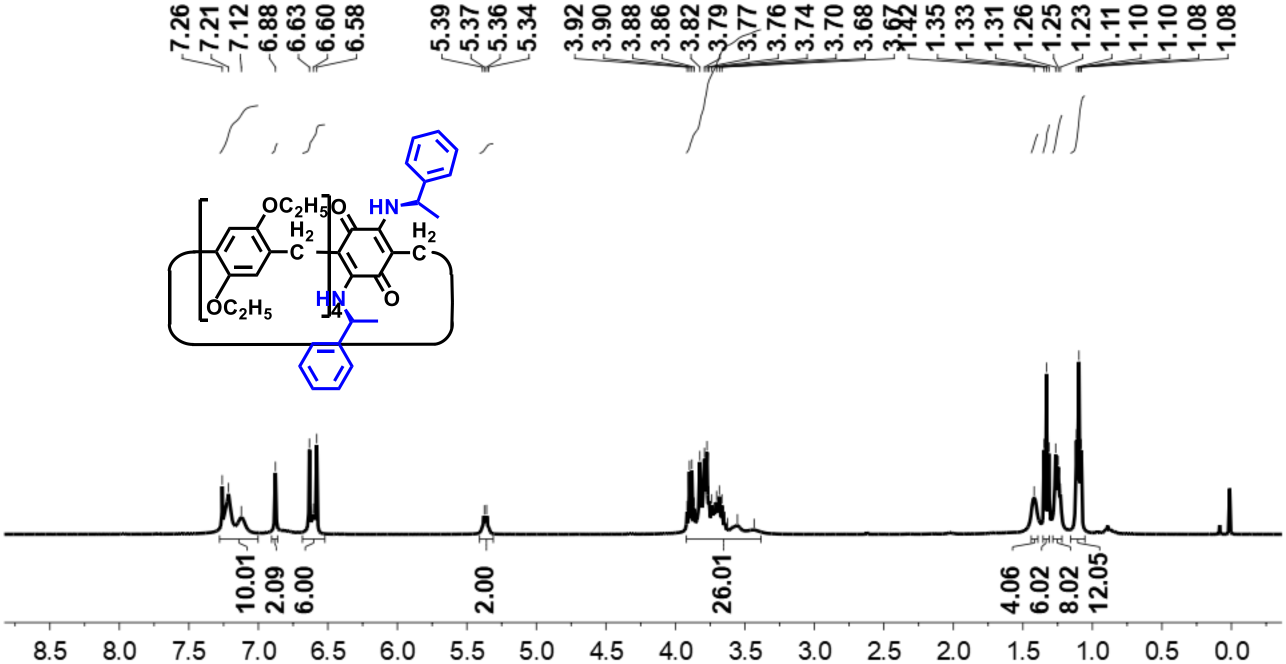

Figure S1. ^{1}H NMR spectrum (400 MHz, $CDCl_3$, 298 K) of compound **1a**.

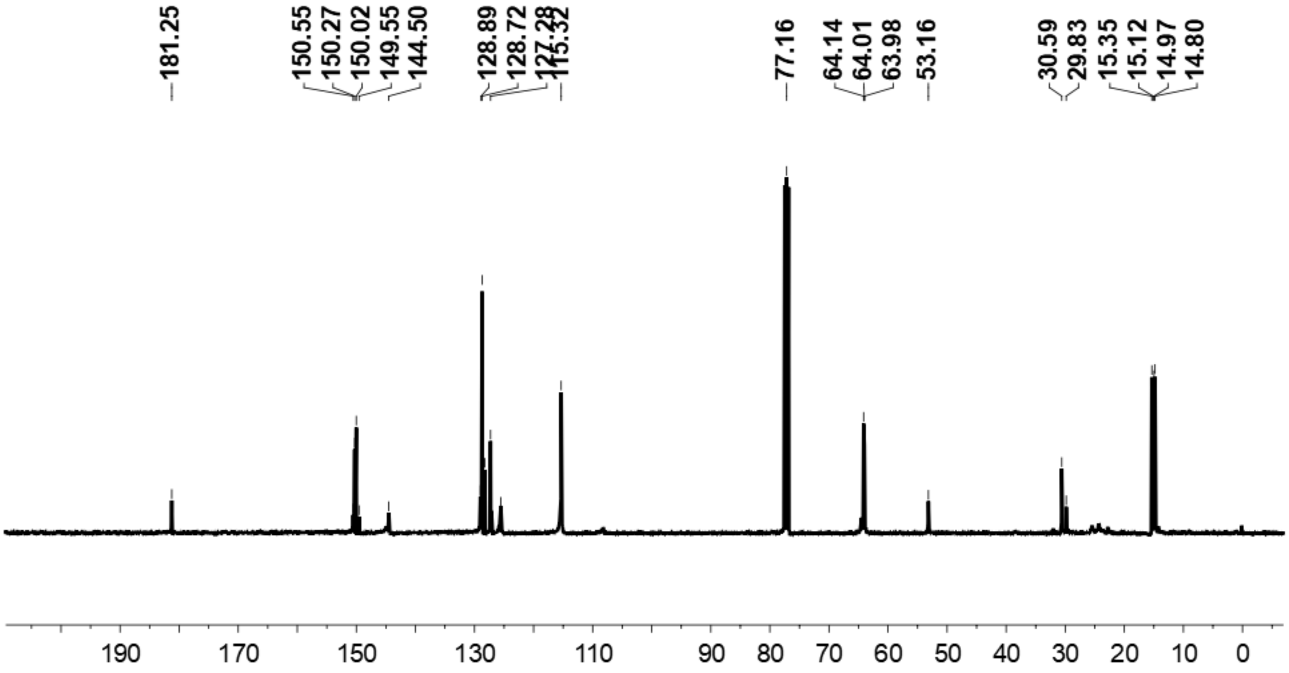

Figure S2. ^{13}C NMR spectrum (100 MHz, $CDCl_3$, 298 K) of compound **1a**.

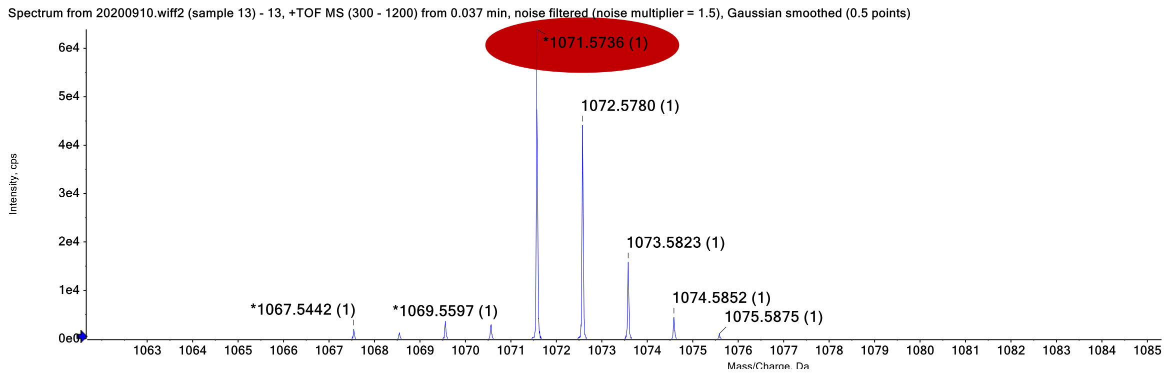

Figure S3. ESI-MS spectrum of **1a**.

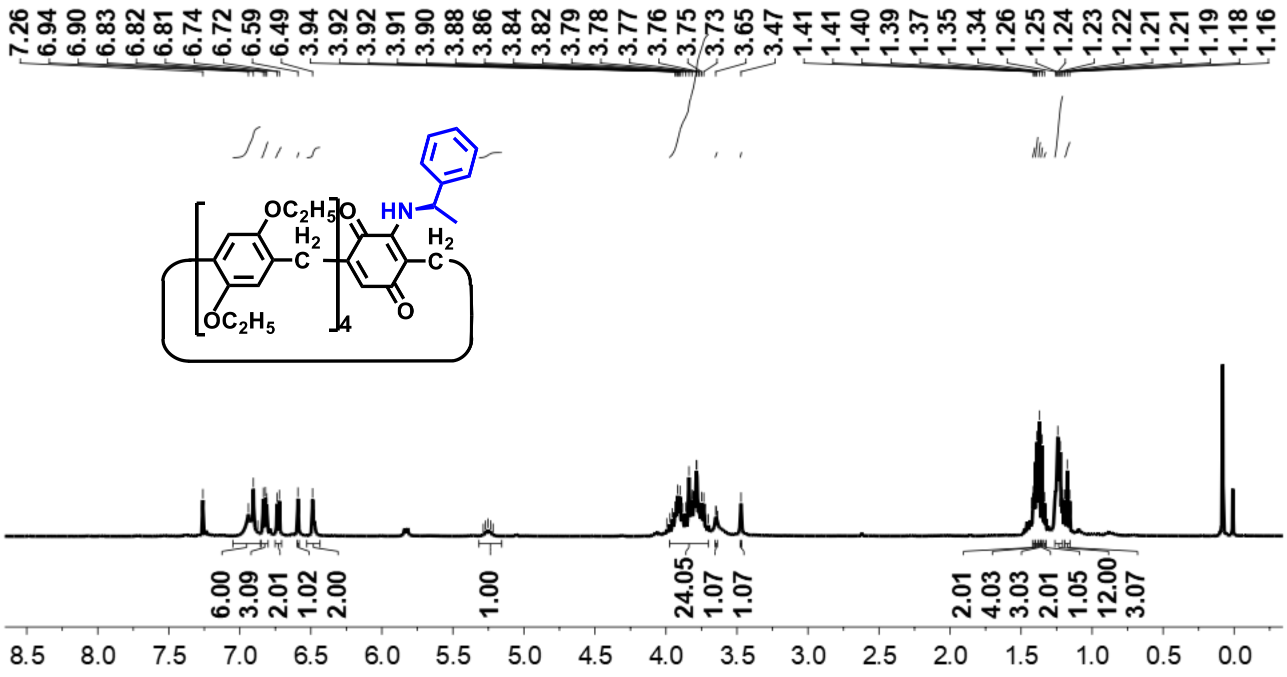

Figure S4. ^{1}H NMR spectrum (400 MHz, $CDCl_3$, 298 K) of **1b**.

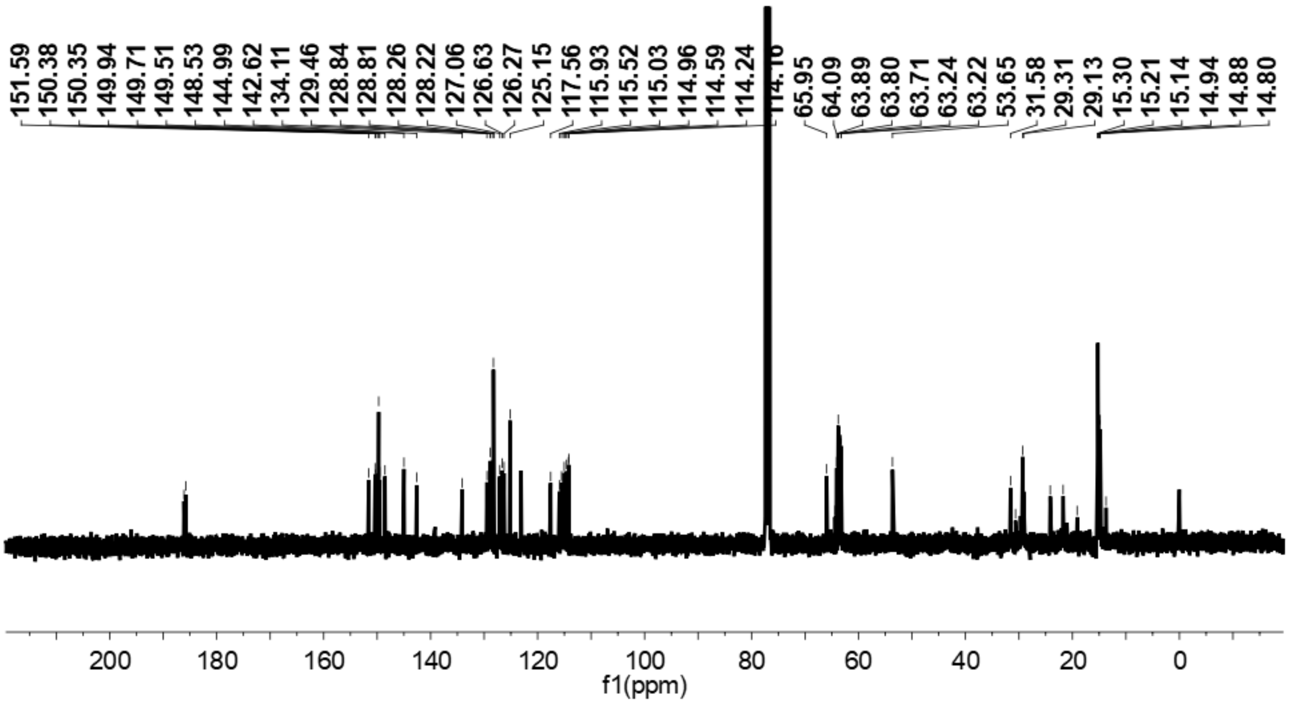

Figure S5. ^{13}C NMR spectrum (100 MHz, $CDCl_{3,}$ 298 K) of compound **1b**.

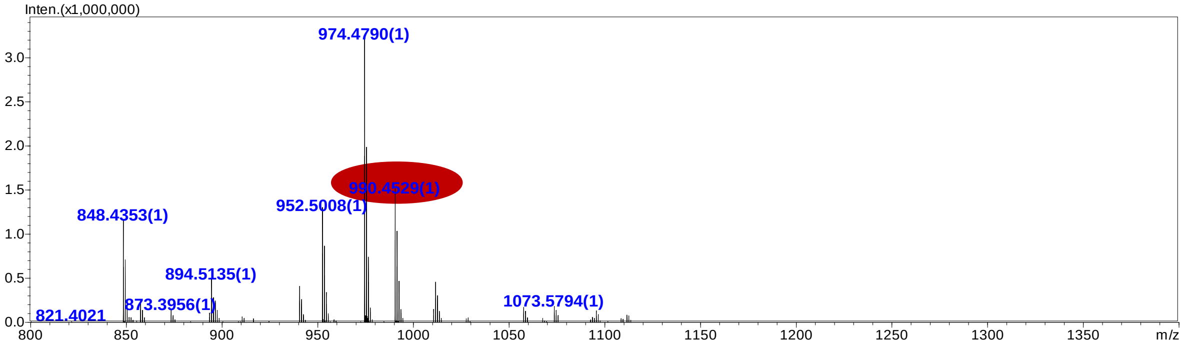

Figure S6. ESI- MS spectrum of **1b**.

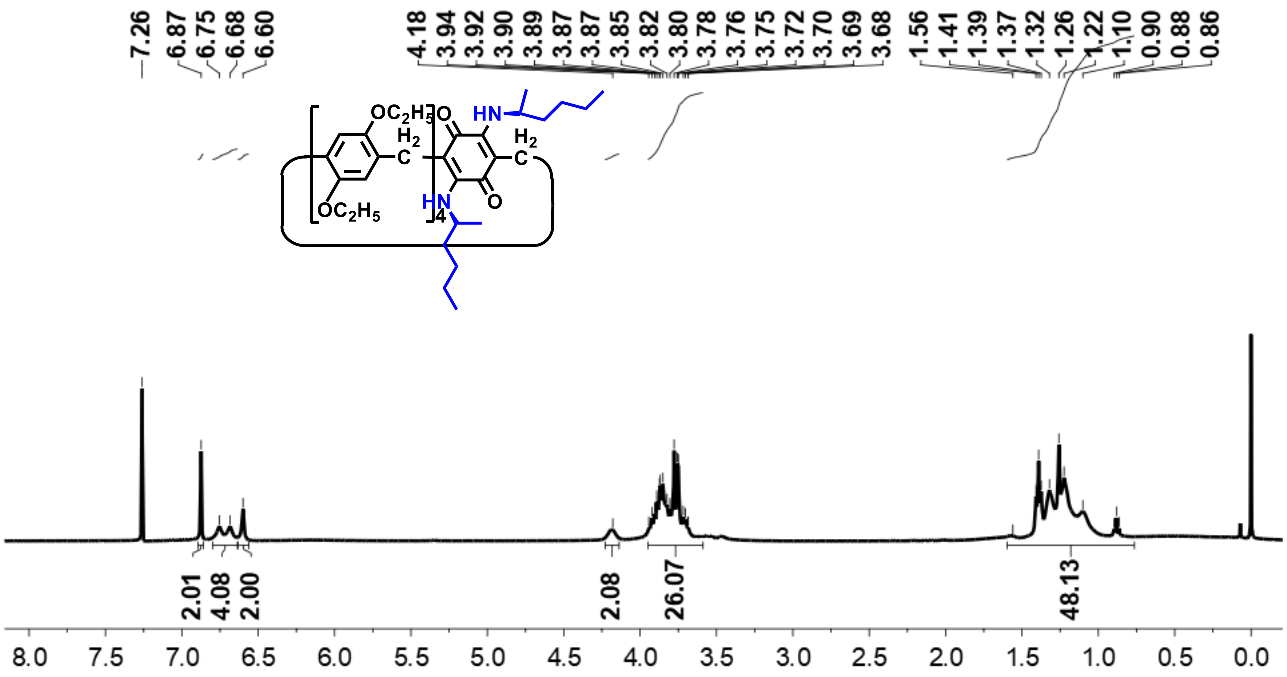

Figure S7. ^{1}H NMR spectrum (400 MHz, $CDCl_3$, 298 K) of compound **2a**.

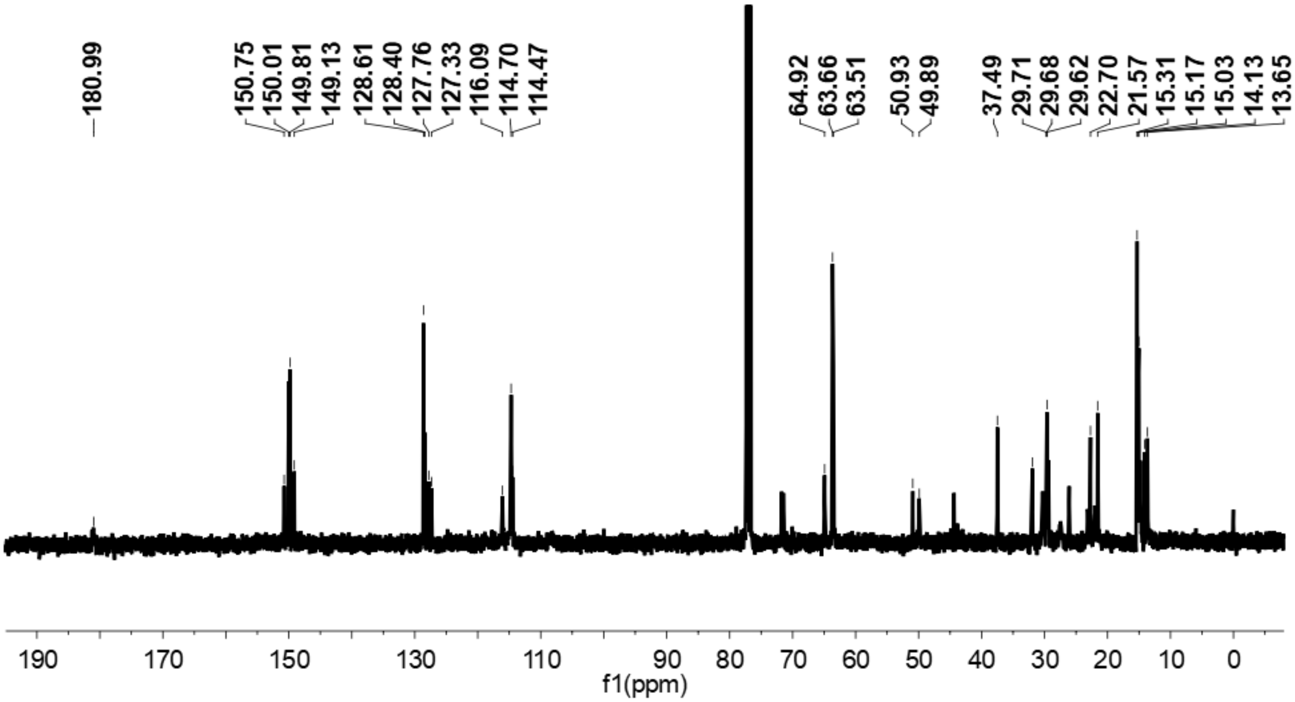

Figure S8. ^{13}C NMR spectrum (100 MHz, $CDCl_3$, 298 K) of compound **2a**.

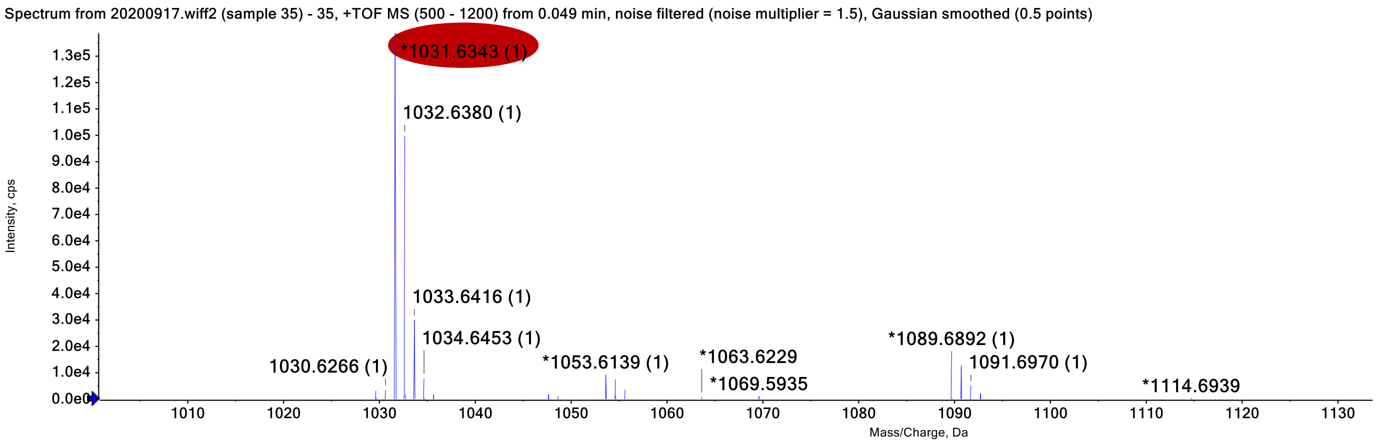

Figure S9. ESI- MS spectrum of **2a**.

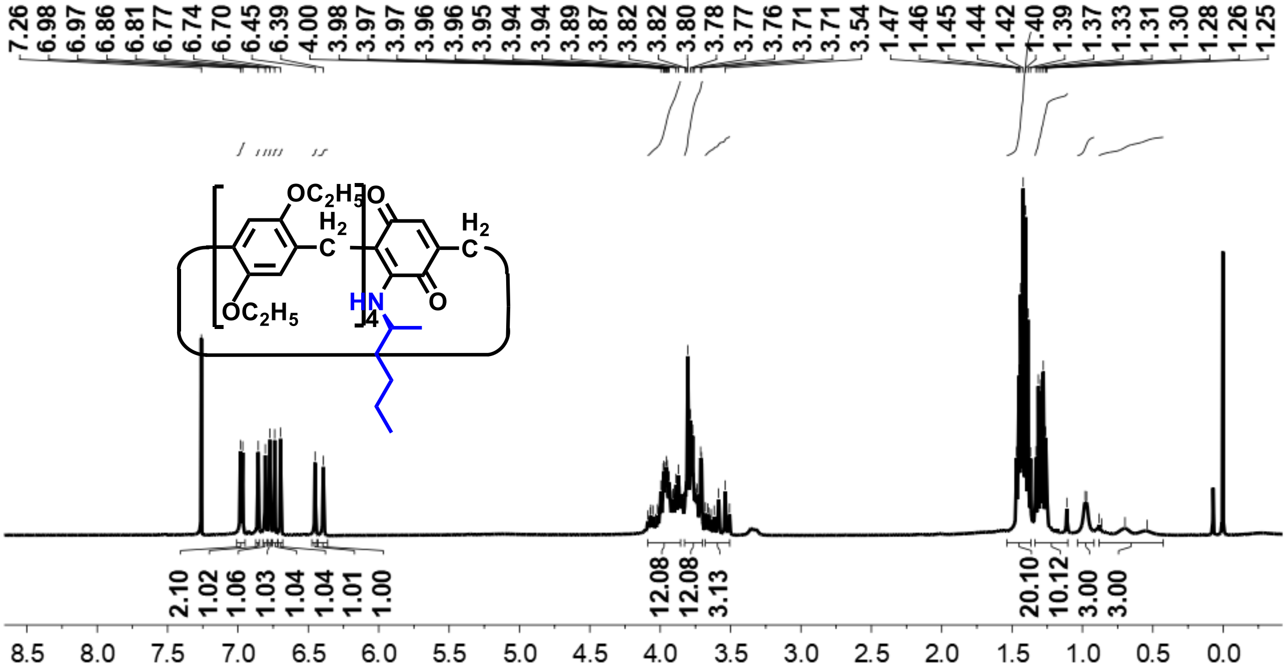

Figure S10. ^{1}H NMR spectrum (400 MHz, $CDCl_3$, 298 K) of **2b**.

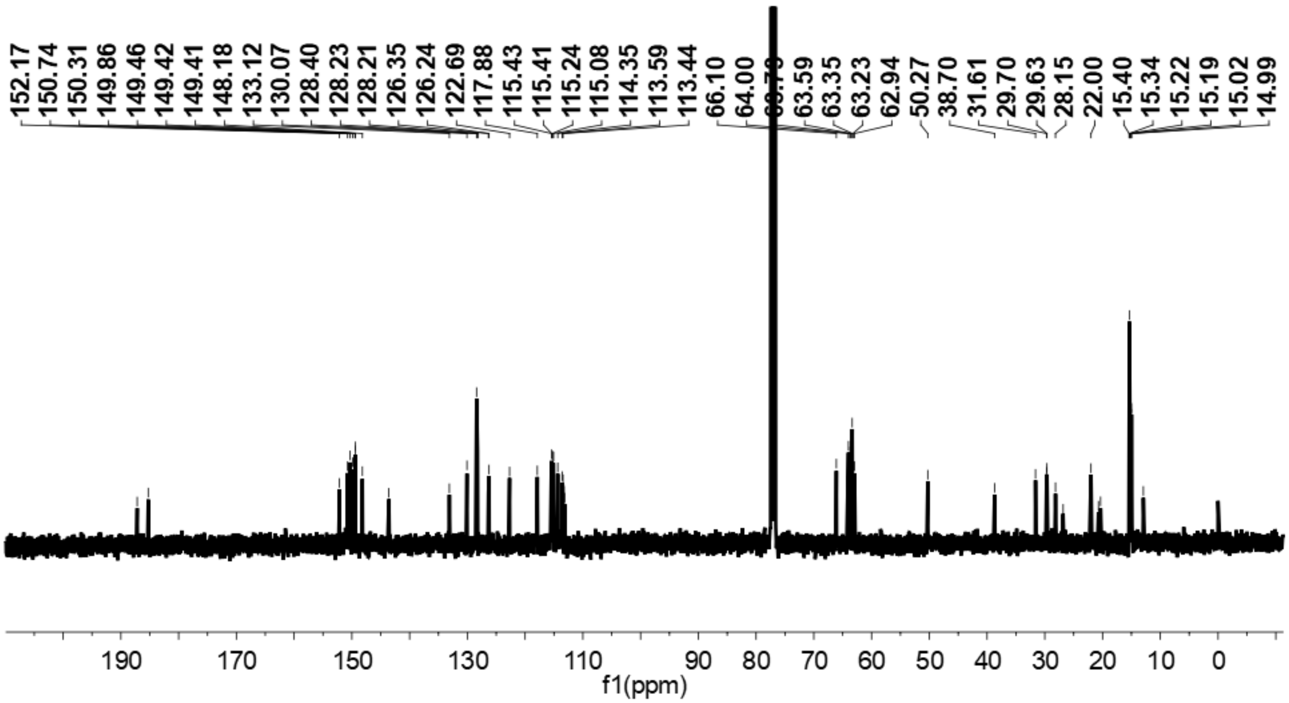

Figure S11. ^{13}C NMR spectrum (100 MHz, $CDCl_3$, 298 K) of compound **2b**.

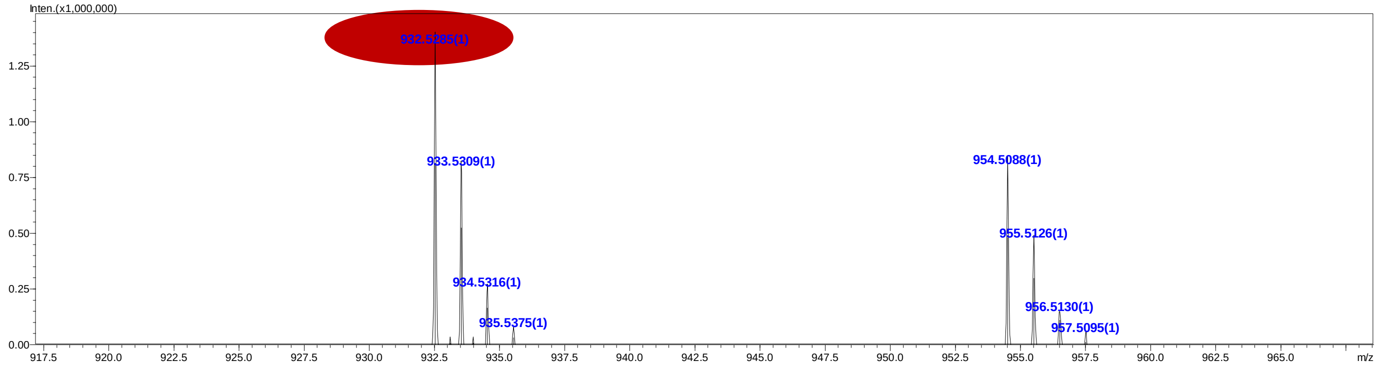

Figure S12. ESI- MS spectrum of **2b**.

UV-vis spectra of 1a

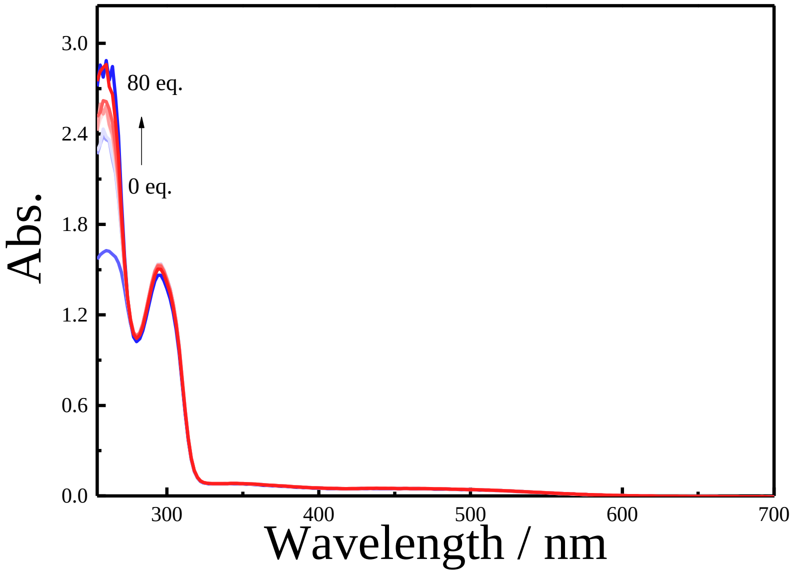

Figure S13. UV-vis spectral changes of pillar[4]arene[1]quinone (**EtP4Q1**) (50 μM in $CHCl_3$) upon the addition of (R)-(+)-α-methylbenzylamine at 25 °C.

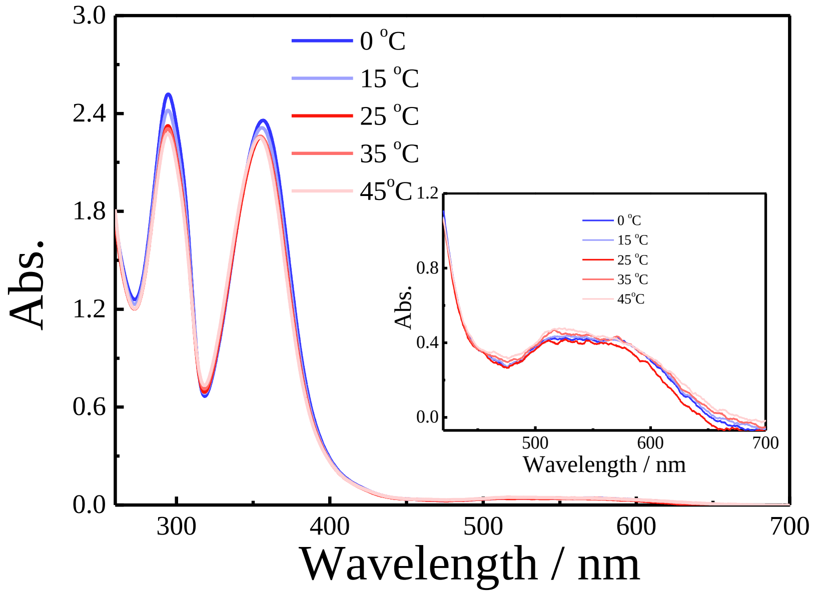

Figure S14. UV-vis absorption spectra of **1a** (100 μM) in chloroform solution at different temperature.

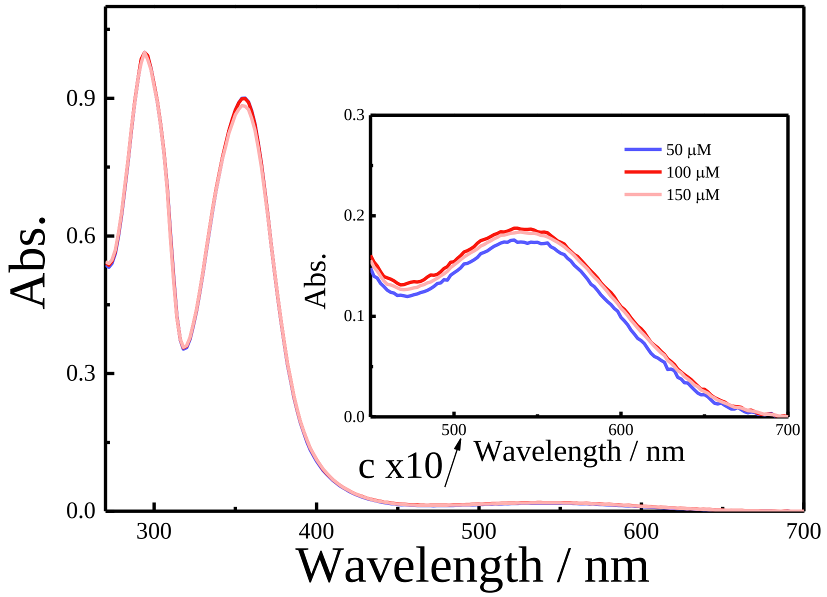

Figure S15. UV-vis absorption spectra of **1a** compound at various concentrations in chloroform solution.

Variable Temperature CD Spectra of 1a

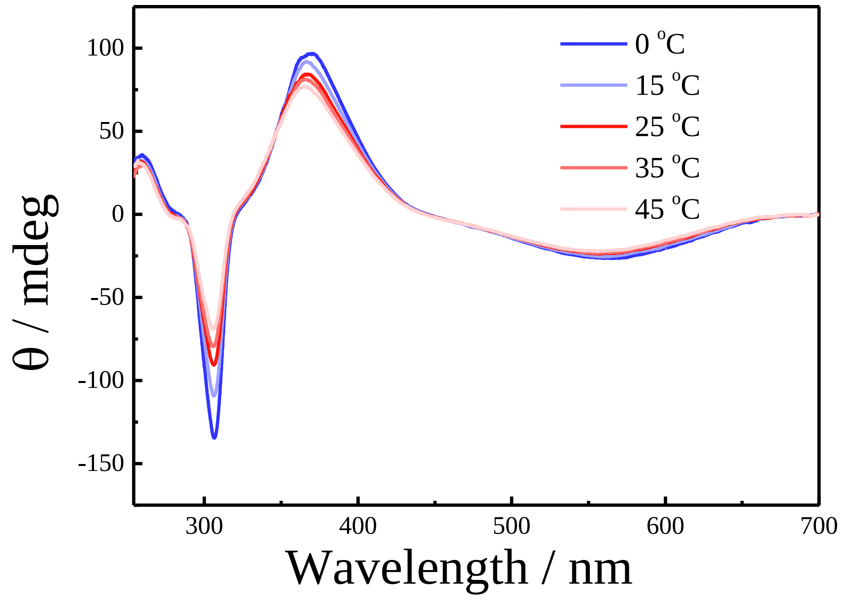

Figure S16. Temperature-dependent CD spectra of **1a** (100 μM) in chloroform.

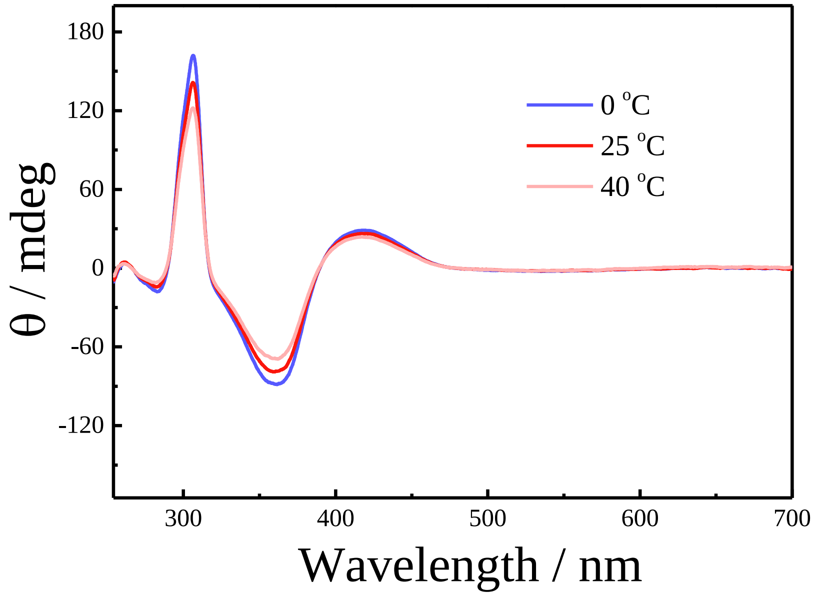

Figure S17. Temperature-dependent CD spectra of **1a** (100 μM) in 1, 2-dichloroethane.

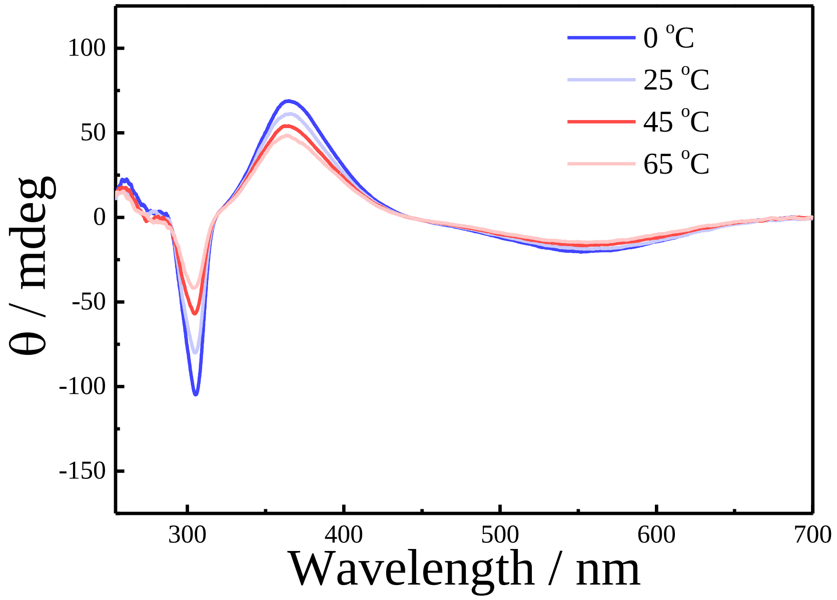

Figure S18. Temperature-dependent CD spectra of **1a** (100 μM) in methanol.

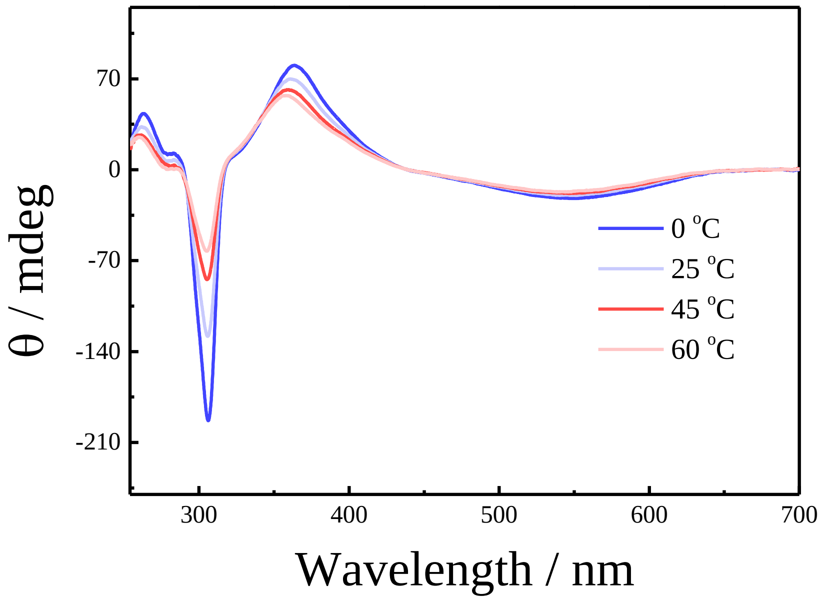

Figure S19. Temperature-dependent CD spectra of **1a** (100 μM) in THF.

Solvent-dependent CD Spectra of 1b, 2a and 2b

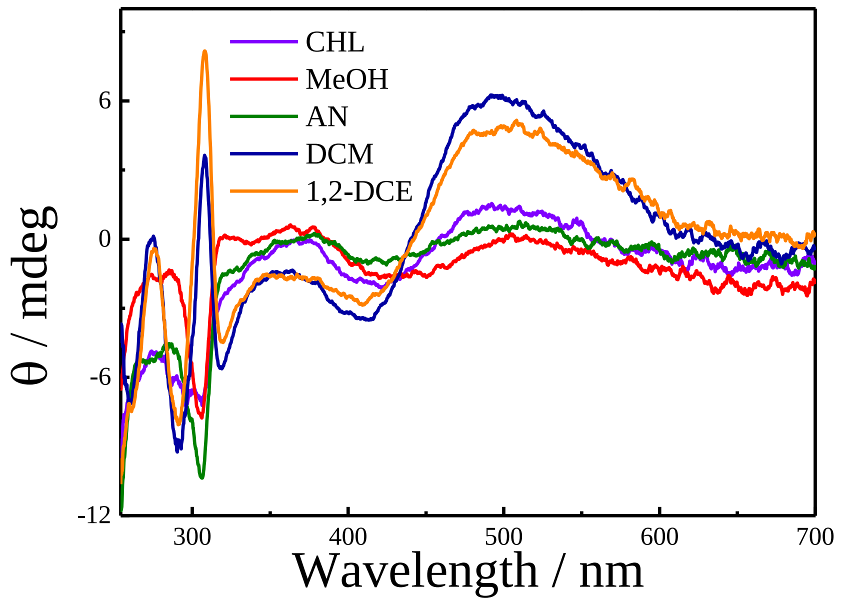

Figure S20. CD spectra of **1b** (100 μM) in various solvents at 25 °C. DCM: dichloromethane, 1,2-DCE:1,2-dichloroethane, CHL: chloroform, MeOH: methanol, AN:acetonitrile.

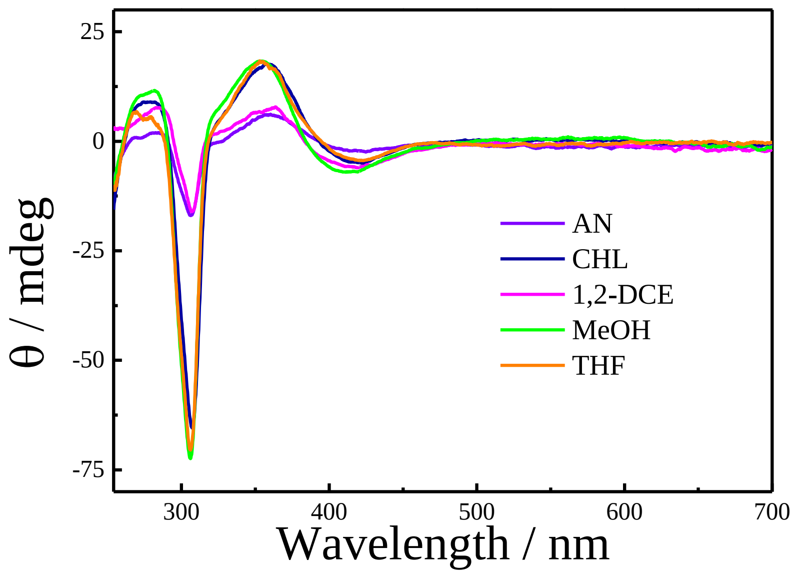

Figure S21. CD spectra of **2a** (100 μM) in various solvents at 25 °C.

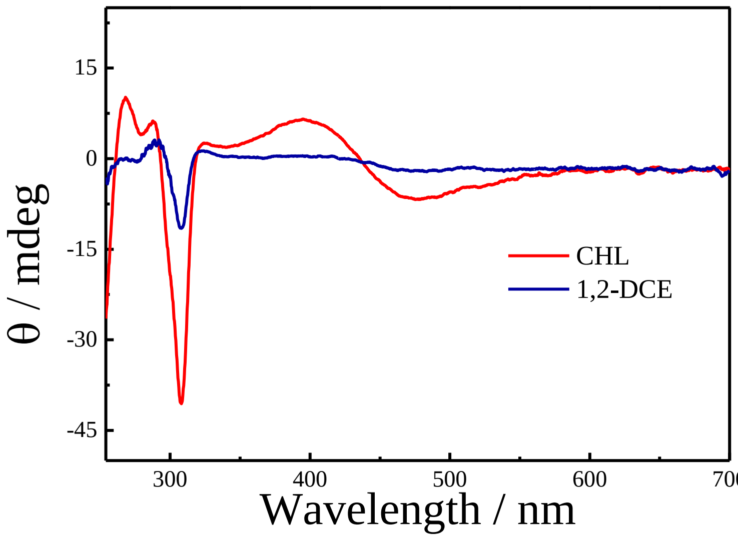

Figure S22. CD spectra of **2b** (100 μM) in various solvents at 25 ^{o}C.

Spectroscopic studies of host and guest binding complexes

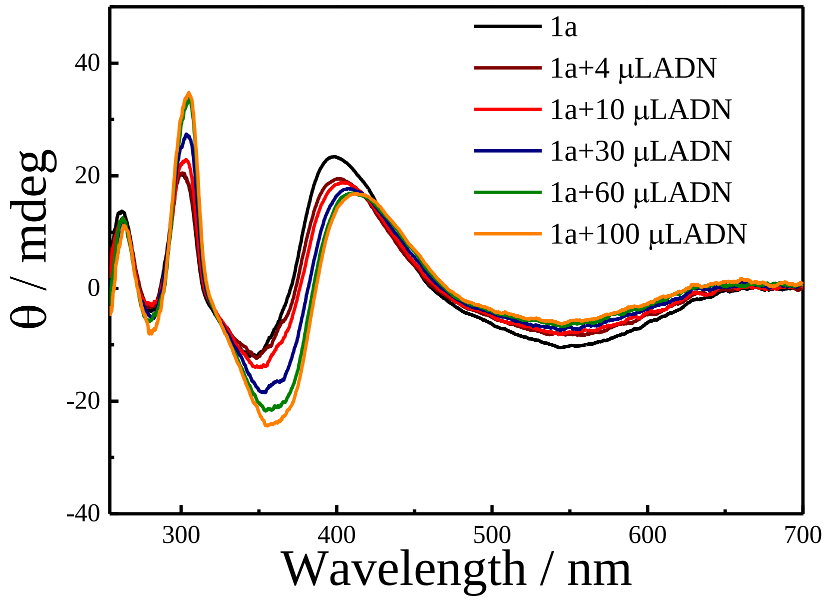

Figure S23. CD spectra changes of **1a** (50 μM in DCM) upon the incremental addition of 1,4-dicyanobutane (1,4-DCB) at 25 ^{o}C.

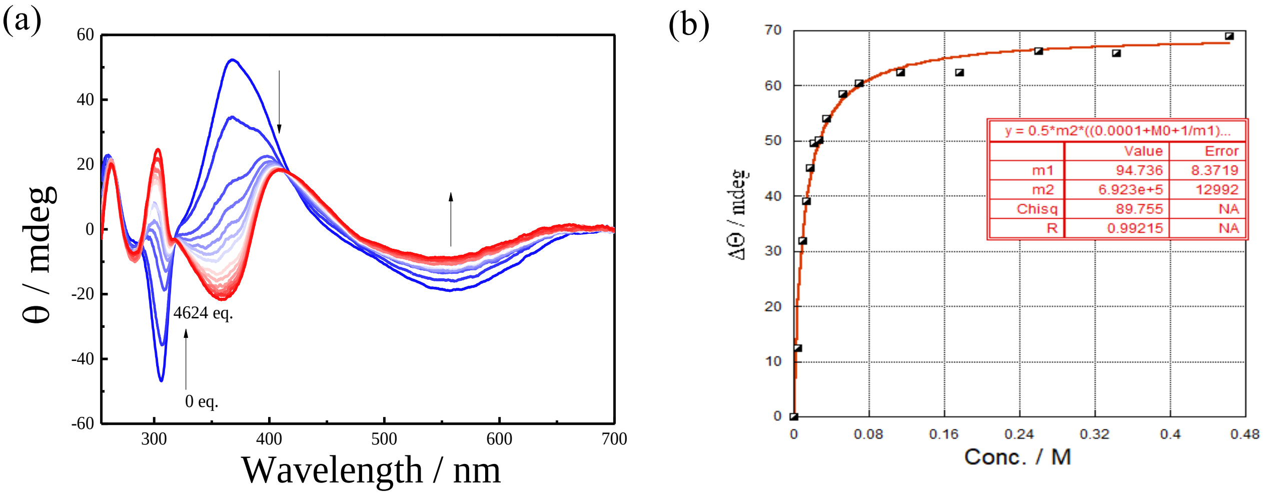

Figure S24. (a) CD spectral changes of **1a** (1.0×10^{-4} M) upon titration with 1,4-DCB (0.46 M) in $CHCl_3$ at 25 °C; (b) The non-linear curve-fitting (CD titrations) for the complexation of 1,4-DCB with **1a** (304 nm) in $CHCl_3$ at 25 ^{0}C, the association constants (Ka) for the complexes is 94.736 (±8.3719) M^{-1}.

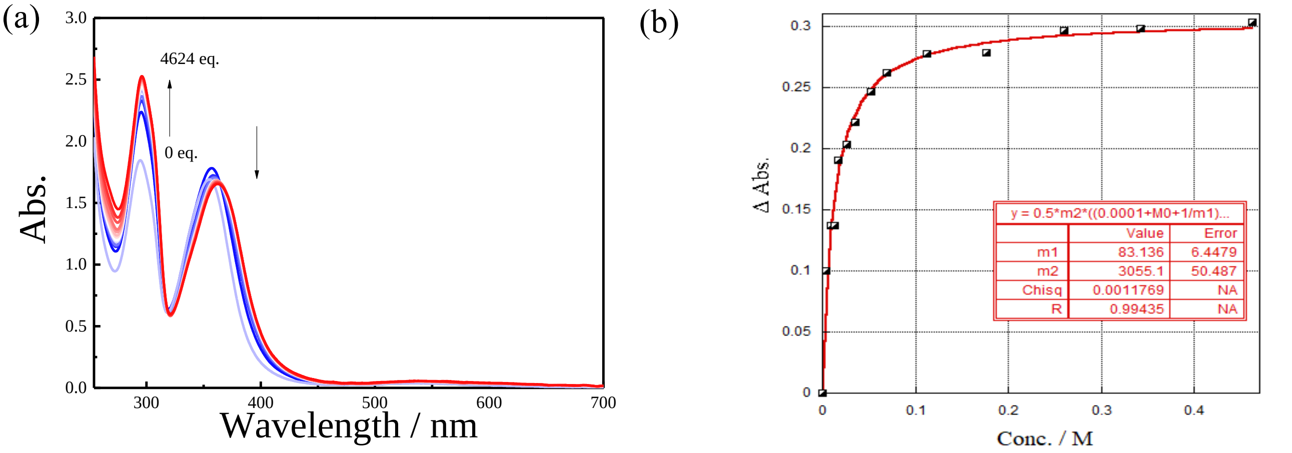

Figure S25. (a) UV/Vis absorption spectra changes of **1a** (1.0×10^{-4} M) upon titration with 1,4-DCB (0.46 M) in $CHCl_3$ at 25 °C; (b) The non-linear curve-fitting (UV titrations) for the complexation of 1,4-DCB with **1a** (298 nm) in $CHCl_3$ at 25 ^{o}C, the association constants (Ka) for the complexes is 83.136 (±6.4479) M^{-1} .

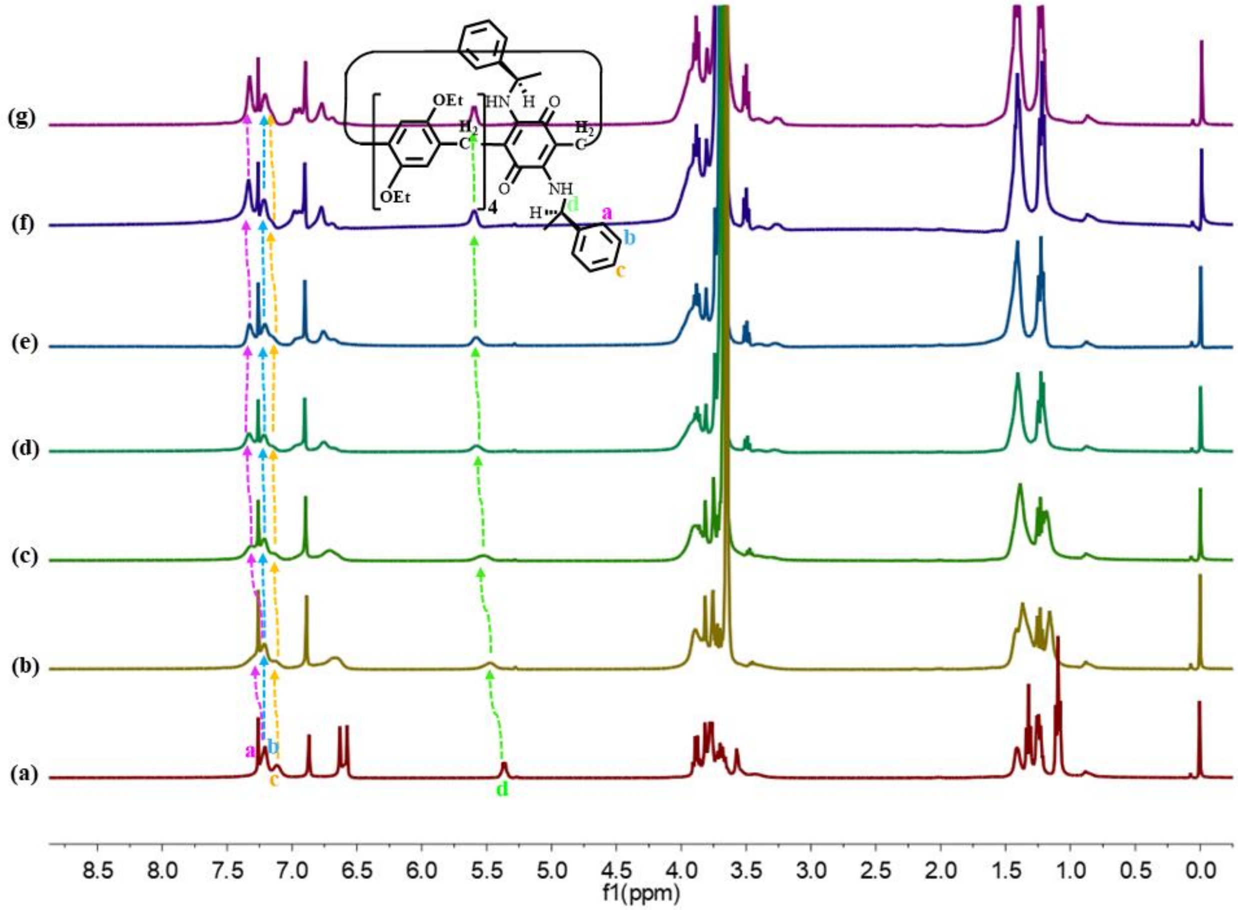

Figure S26. ^{1}HNMR spectra of **1a** (a) at $CDCl_3$; (b) at $CDCl_3$:1.2-DCE = 50:1; (c) at $CDCl_3$:1.2-DCE = 25:1; (d) at $CDCl_3$:1.2-DCE = 25:2; (e) at $CDCl_3$:1.2-DCE = 10:1; (f) at $CDCl_3$:1.2-DCE = 50:7; (g) at $CDCl_3$:1.2-DCE = 50:9; (v/v) (400 MHz, 298 K).

Reference

Han, C., Zhang, Z., Yu, G., and Huang, F. (2012). Syntheses of a pillar[4]arene[1]quinone and a difunctionalized pillar[5]arene by partial oxidation. *Chem. Commun*. 48, 9876-9878. doi: 10.1039/C2CC35498E.

Ogoshi, T., Kitajima, K., Aoki, T., Fujinami, S., Yamagishi, T.-a., and Nakamoto, Y. (2010). Synthesis and Conformational Characteristics of Alkyl-Substituted Pillar[5]arenes. *J. Org. Chem.* 75, 3268-3273. doi: 10.1021/jo100273n.
